# Prognostic impact of immunohistochemical expression of CK7 and CK20 in curatively resected ampulla of Vater cancer

**DOI:** 10.1186/s12876-015-0396-x

**Published:** 2015-11-24

**Authors:** Sung Pil Yun, Hyung Il Seo

**Affiliations:** Department of surgery, Biomedical Research Institute, Pusan National University Hospital, 189 Gudeok-Ro, Seo-Gu, Busan, 602-739 South Korea

**Keywords:** Ampulla of Vater adenocarcinoma, Prognostic factor, Immunohistochemical expression, CK7, CK20

## Abstract

**Background:**

In the consideration of ampullary adenocarcinoma, T stage, lymph node metastases, perineural invasion, tumor differentiation, pancraticobiliary type, and lymph node ratio are considered prognostic factors. The objectives of this study were to investigate surgical outcomes and the clinicopathological predictors affecting survival and recurrence, and to examine the prognostic roles of histopathological subtype and immunohistochemical markers.

**Methods:**

From April 2006 to September 2012, 37 patients who underwent curative resection of ampullar of Vater adenocarcinoma were enrolled in this study. A retrospective review was performed based on medical records. Immunohistochemical expression, histopathological type and clinicopathologic factors were analyzed.

**Results:**

The 5-year overall survival rates and disease-free survival rates after surgery were 77.4 and 75.7 %, respectively. Multivariate Cox regression analysis showed that advanced T stage (*p* = 0.019) and positive expression of Cytokeratin 7 (CK7) with negative expression of Cytokeratin 20 (CK20) (*p* = 0.046) were identified as significant independent factors related to survival, and poor differentiation (*p* = 0.031) significantly influenced disease-free survival in multivariate analysis.

**Conclusions:**

Advanced T stage is a significant prognostic factor affecting survival in ampullary adenocarcinoma. Also, positive expression of CK7 with negative expression of CK20 is an independent factor related to overall survival.

## Background

Adenocarcinoma of the ampulla of Vater (AoV) is a relatively rare neoplasm accounting for 0.5 % of all gastrointestinal malignancies and about 30 % of all cases requiring pancreaticoduodenectomy [1–3]. Adenocarcinoma of the AoV is a type of periampullary carcinoma with a better prognosis than other periampullary carcinomas [4, 5]. Compared with other tumors, ampullary adenocarcinomas tend to be detected relatively early. Therefore, ampullary adenocarcinomas have a higher resection rate at the time of diagnosis than other periampullary carcinoma, and they have a better prognosis compared with pancreatic and bile duct malignancies [5–7].

AoV adenocarcinomas can be categorized into two major histologic types. Intestinal type adenocarcinomas of the duodenal papilla originate from the intestinal mucosa covering the papilla and evolve through an adenoma-dysplasia-carcinoma sequence. Pancreaticobiliary type adenocarcinomas are derived from ductal epithelium that penetrates the duodenal muscularis propria containing the distal common bile duct, the distal pancreatic duct, or the common ampullary channel [8, 9]. Apparently, pancreaticobiliary type ampullary adenocarcinoma is associated with lower survival rates [10, 11]. Cytokeratin 7 (CK7) and Cytokeratin 20 (CK20) are reliable and well-characterized immunohistochemical markers, and are usually helpful in distinguishing intestinal type and pancreaticobiliary type adenocarcinoma. According to a previous study, CK7 was expressed in 91.4 % of the pancreaticobiliary type ampullary carcinomas, and CK20 expression was positive in about 90 % of the intestinal type ampullary carcinomas, but was generally negative in the pancreaticobiliary type [10]. However, the expression profiles of CK7 and CK20 in relation to tumor histotype and prognosis have not been carefully investigated in ampullary adenocarcinomas. The objectives of this study were to investigate surgical outcomes, the clinicopathological predictors affecting survival and recurrence, and the prognostic roles of histopathological subtype and immunohistochemical markers of resected ampullary adenocarcinoma.

## Methods

### Patients

**Fig. 1:**
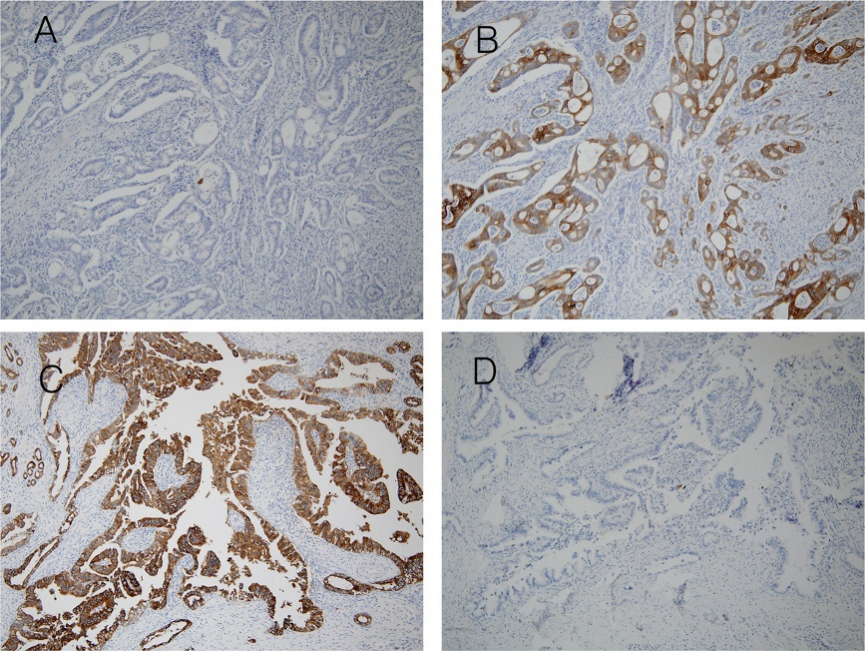
Cytokeratin 7 (CK7) and cytokeratin 20 (CK20) expression in ampulla of Vater adenocarcinoma. **a**, **b** CK7 and CK 20 in one patient displaying intestinal subtype adenocarcinoma. **a** CK7 negative and **b** CK20 positive. **c**, **d** one patient displaying pancreaticobiliary type adenocarcinoma. **c** CK7 positive and **d** CK20 negative

Patients who underwent curative resection of AoV adenocarcinoma at Pusan National University Hospital from April 2006 to September 2012 were enrolled in this study. A retrospective review was performed based on medical records. Patients who underwent R1 resection or palliative resection and patients with stage IV disease were excluded, as well as patients with other types of carcinomas. Finally, 37 patients were included. This retrospective study was approved by the institutional review board at Pusan National University Hospital Clinical Trial Center (IRB No.2015053), and written informed consent was obtained from all participants. Twenty-eight patients underwent pylorus preserving pancreaticodoudenectomy, and 9 patients underwent conventional pancreaticoduodenectomy. The following clinical information was retrospectively reviewed from patient records: sex, age, T stage, lymph node metastases, AJCC stage, lymphovascular invasion, perineural invasion, tumor size, tumor differentiation, preoperative carbohydrate antigen 19–9 (CA 19–9), preoperative carcinoembryonic antigen (CEA), postoperative pancreatic fistula, adjuvant chemotherapy or radiotherapy, histopathological subtype, and immunohistochemical markers. Histological diagnosis was examined by a pathologist who specializes in hepatobiliary pathology. Immunohistochemistry for these antibodies were performed on formalin-fixed embedded sections on the fully automated Bond Max automatic slide stainer (Leica Microsystems, Bannockburn, IL, USA). Primary antibodies were prediluted monoclonal antibodies clone against CK7 and CK20 proteins (CK7 clone OV-TL12/30; CK20 PW31). Classification of adenocarcinoma into intestinal or pancreaticobiliary types was performed using cytologic and architectural features according to criteria revised by Albores-Saavedra et al. [12]. CK7 and CK20 expression was established using the percentage of immunoreactive cells calculated by the number of immunoreactive cells over the total number of tumor cells. Cases showing greater than 5 % tumor cell positivity were regarded as positive (Fig. 1) [8].

### Statistical analysis

We analyzed clinicopathological features, disease-free survival, and overall survival rates. Overall survival rate was measured from the date of surgery to the date of death from any cause; locoregional recurrences, distant metastases and second primary cancer were ignored. Disease-free survival was measured from the date of surgery to the date of second cancer, locoregional recurrence, distant metastases or death from any cause. Categorical variables were compared using the chi-square test or Fisher’s exact test. Overall survival and disease-free survival were estimated according to the Kaplan-Meier method, and survival differences were evaluated using the log-rank test. Both univariate and multivariate Cox proportional hazards regression models were used to identify risk factors for recurrence or death. Risk factors in univariate models were included in multivariate models. *P* values less than 0.05 were considered statistically significant.

## Results

Clinicopathological features are summarized in Table 1. The cohort consisted of 37 patients with pathological diagnoses of radically resected adenocarcinoma of the ampulla of Vater. The median age of the patients was 63 years old (range: 47–80 years), and 19 of the patients were females (51.4 %). Fourteen patients (37.8 %) were classified as T1, 8 patients (21.6 %) were classified as T2, 14 patients (37.8 %) were classified as T3, and 1 patient (2.7 %) was classified as T4. Nodal metastasis was present in 6 (16.2 %) of the tumor specimens. The incidence of T1 or T2 AoV adenocarcinoma was slightly high. Tumor size ranged from 0.9 to 7.1 cm and averaged 2.58 ± 1.49 cm. Lymphovascular invasion was found in 6 patients (16.2 %), and 7 tumors (18.9 %) showed perineural invasion. 6 tumors were poorly differentiated. 17 patients underwent adjuvant therapy (chemotherapy or concurrent chemoradiotherapy)

**Table 1:** Demographics and clinical characteristics of patients with ampullary adenocarcinoma

Variables	
Total number	37 (100 %)
Sex	
Male	18 (48.6 %)
Female	19 (51.4 %)
Age, years	62.57 ± 8.22
T stage	
T1	14 (37.8 %)
T2	8 (21.6 %)
T3	14 (37.8 %)
T4	1 (2.7 %)
N stage	
N0	31 (83.8 %)
N1	6 (16.2 %)
Overall stage	
IA	14 (37.8 %)
IB	6 (16.2 %)
IIA	11 (29.7 %)
IIB	5 (13.5 %)
III	1 (2.7 %)
Tumor size (cm)	2.58 ± 1.49
CA19-9 (U/ml)	459.54 ± 1727.30
CEA (ng/ml)	4.03 ± 5.65
Lymphovascular invasion	
No	31 (83.8 %)
Yes	6 (16.2 %)
Perineural invasion	
No	30 (81.1 %)
Yes	7 (18.9 %)
Differentiation	
Well/Moderately	31 (83.8 %)
Poorly	6 (16.2 %)
Adjuvant therapy	
No	20 (54.0 %)
Yes	17 (46.0 %)

*CA19-9* Carbohydrate antigen 19–9, *CEA* Carcinoembryonic antigen

**Fig. 2:**
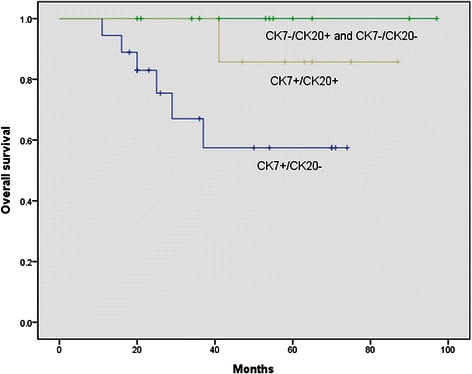
Overall survival in patients with resected ampulla of Vater adenocarcinoma according to immunohistochemical expression

The median duration of follow-up after surgery was 41 months (range, 11–97). The 5-year overall survival rate after surgery was 77.4 %. The 5-year disease-free survival rate was 75.7 %. CK7 positive and CK20 negative (CK7+/CK20-) was significantly more common in the pancreaticobiliary type than in the intestinal type according to the chi-square test (*p* = 0.005). Sex, age, T stage, lymph node metastases, AJCC stage, lymphovascular invasion, perineural invasion, tumor size, tumor differentiation, CA 19–9, CEA, and postoperative pancreatic fistula were similar for CK7+/CK20- and non-CK7+/CK20- patients. By univariate analysis, overall survival in this study was found to be influenced significantly by advanced T stage (T3 or T4) (*p* = 0.015), lymph node metastases (*p* = 0.021), positive lymphvascular invasion (*p* = 0.024), positive perineural invasion (*p* = 0.041), poor differentiation (*p* = 0.013), AJCC stage (*p* = 0.021) and CK7+/CK20- (*p* = 0.036) (Table 2). Figure 2 shows overall survival using a Kaplan-Meier survival plot in patients with resected ampulla of Vater adenocarcinoma according to immunohistochemical expression. Multivariate Cox regression analysis of factors identified by univariate analysis showed that advanced T stage and CK7+/CK20- were identified as significant independent factors related to survival (Table 3). In terms of disease-free survival, univariate analysis showed that advanced T stage (T3 or T4) (*p* = 0.011), lymph node metastases (*p* = 0.010), positive lymphvascular invasion (*p* = 0.009), positive perineural invasion (*p* = 0.019), poor differentiation (*p* < 0.001), AJCC stage (*p* = 0.010), pancreaticobiliary type (*p* = 0.046) and adjuvant therapy (*p* = 0.042) significantly influenced recurrence (Table 2). Multivariate analysis of factors identified by univariate analysis showed that poor differentiation (*p* = 0.031) significantly influenced disease-free survival (Table 4).

**Table 2:** Univariate analysis for predictive factors influencing overall survival and disease-free survival after curative resection

Variables	N	5 years survival rate	Overall survival		5 years survival rate	Disease-free survival	
			Hazard ratio	*P* value		Hazard ratio	*P* value
Sex							
Male	18	87.2	2.33 (0.45–12.05)	0.311	77.8	1.07 (0.28–3.99)	0.919
Female	19	68.0			73.7		
Age, years							
< 60	14	80.0	1.79 (0.34–9.21)	0.489	78.6	1.27 (0.32–5.09)	0.733
≥ 60	23	75.9			73.9		
Subtype							
Intestinal	17	93.3	8.45 (0.99–71.61)	0.051	94.1	8.34 (1.04–66.88)	0.046
Pancreaticobiliary	20	60.5			60.0		
CK7 +/CK20 -							
No	19	93.3	9.74 (1.16–81.89)	0.036	89.5	4.31 (0.89–20.77)	0.069
Yes	18	57.5			61.1		
CDX2							
No	20	75.2	1.26 (0.28–5.68)	0.764	75.0	1.02 (0.28–3.81)	0.972
Yes	16	79.3			75.0		
T stage							
T1, T2	22	95.2	13.94 (1.66–117.03)	0.015	95.5	14.81 (1.85–118.92)	0.011
T3, T4	15	45.4			46.7		
Lymph node metastasis							
No	31	88.4	6.00 (1.30–27.58)	0.021	83.9	5.69 (1.52–21.30)	0.010
Yes	6	33.3			33.3		
Lymphovascular invasion							
No	31	85.1	5.89 (1.27–27.38)	0.024	83.9	6.06 (1.58–23.28)	0.009
Yes	6	31.3			33.3		
Perineural invasion							
No	30	84.0	4.83 (1.07–21.87)	0.041	83.3	4.88 (1.29–18.40)	0.019
Yes	7	47.6			42.9		
Size							
< 2 cm	14	83.1	1.92 (0.37–9.94)	0.436	85.7	2.34 (0.49–11.25)	0.290
≥ 2 cm	23	73.8			69.6		
Differentiation							
Well/mod	31	86.0	8.18 (1.57–42.67)	0.013	87.1	12.29 (3.06–48.31)	<0.001
Poorly	6	0			16.7		
Stage							
I and IIA	31	88.4	6.00 (1.30–27.58)	0.021	83.9	5.69 (1.52–21.30)	0.010
IIB and III	6	33.3			33.3		
Ca19-9 (U/ml)							
< 37	12	70.0	0.66 (0.13–3.28)	0.611	66.7	0.40 (0.09–1.81)	0.236
≥ 37	20	84.0			85.0		
CEA (ng/ml)							
< 5	27	83.2	1.28 (0.14–11.54)	0.826	81.5	1.37 (0.16–11.74)	0.774
≥ 5	4	75.0			75.0		
Pancreatic fistula							
No	21	77.2	0.88 (0.20–3.95)	0.872	71.4	0.59 (0.15–2.35)	0.453
Yes	16	77.9			81.3		
Adjuvant therapy							
No	20	90.0	3.67 (0.70–19.02)	0.122	90.0	5.11 (1.06–24.68)	0.042
Yes	17	58.2			58.8		

*CK7* Cytokeratin 7, *CK20* Cytokeratin 20, *CA19-9* Carbohydrate antigen 19–9, *CEA* Carcinoembryonic antigen

**Table 3:** Multivariate analysis of overall survival in patients with ampullary adenocarcinoma

Variables	Hazard ratio	*P* value
CK7+/CK20-		
No	Ref.	0.046
Yes	8.96 (1.04–77.38)	
T stage		
T1, T2	Ref.	0.019
T3, T4	12.98 (1.52–110.78)	
Lymph node metastasis		
No	Ref.	0.276
Yes	2.45 (0.49–12.33)	
Lymphovascular invasion		
No	Ref.	0.678
Yes	0.67 (0.10–4.55)	
Differentiation		
Well/mod	Ref.	0.955
poorly	1.05 (0.17–6.59)	

*CK7* Cytokeratin 7, *CK20* Cytokeratin 20

**Table 4:** Multivariate analysis of disease-free survival in patients with ampullary adenocarcinoma

Variables	Hazard ratio	*P* value
Subtype		
Intestinal	Ref.	0.664
Pancreaticobiliary	1.72 (0.15–19.54)	
T stage		
T1, T2	Ref.	0.063
T3, T4	8.21 (0.89–75.54)	
Lymph node metastasis		
No	Ref.	0.091
Yes	3.35 (0.82–13.63)	
Lymphovascular invasion		
No	Ref.	0.447
Yes	1.97 (0.34–11.39)	
Differentiation		
Well/mod	Ref.	0.031
poorly	4.95 (1.16–21.18)	

## Discussion

Adenocarcinoma of the ampulla of Vater is a relatively rare neoplasm. In contrast to pancreatic cancer, at least 80 % of patients with ampullary adenocarcinoma are candidates for potentially curative resection [13]. Standard surgical therapies include pancreaticoduodenectomy or ampullectomy in patients for whom radical resection is not feasible. High resectability rate and early detection of carcinoma due to early symptom onset, such as obstructive jaundice, are more closely related to a better prognosis in this carcinoma than in distal bile duct carcinoma or pancreatic head carcinoma [5, 9, 14, 15]. According to previous studies, 5-year survival rates have been reported in the range of 38 to 68 % [15–23]. Similarly, in this study, the 5-year survival rate in patients who underwent a radical operation was 77.4 %. Many studies have examined the prognosis of ampullary adenocarcinoma. Several clinicopathological factors have been reported to influence prognosis after curative resection. Previous studies have reported that the depth of tumor infiltration (T stage) is an important prognostic factor [15, 16]. Also, prognostic factors for ampullary adenocarcinoma following curative surgery have been reported as lymph node metastases [15–17, 24], perineural invasion [16, 18, 20, 23], tumor differentiation [18, 25], pancraticobiliary type [9, 11], and lymph node ratio [24, 26]. In this study, our multivariate analysis showed that advanced T stage and CK7+/CK20- were identified as significant independent factors related to survival, and poor differentiation significantly influenced the rate of disease-free survival.

In recent studies, the pancreatobiliary subtype of ampullary adenocarcinomas demonstrated more node metastases and was associated with a poorer prognosis than was observed with the intestinal subtype [9, 11, 27, 28]. However, some studies reported no significant prognostic differences between the intestinal subtype and the pancreaticobiliary subtype [2, 29, 30]. In the present study, overall survival was not found to be significantly shorter for pancraticobiliary subtype ampullary adenocarcinoma in univariate analysis. With regard to immunohistologic results, CK7+/CK20- was identified as a factor influencing survival in multivariate analysis.

Generally, the immunohistochemistry of intestinal type adenocarcinomas is CK7 negative and CK20 positive, whereas the immunohistochemistry of pancreaticobiliary type adenocarcinomas is CK7 positive and CK20 negative, as is pancreatic ductal epithelium [29]. Zhou et al. [29]. Reported a close correlation between tumors characterized by histological features and those characterized by cytokeratin expression. According to a study by Morini et al. [10], of 72 cases, there was a total of 31 cases of the intestinal type and 35 cases of the pancreaticobiliary type. They commented that CK20 was an independent factor related to prognosis, however, there was no significant prognostic difference between the intestinal type and the pancreaticobiliary type. In the present study, we identified a total of 17 cases of the intestinal type and 20 cases of the pancreaticobiliary type. The pancreatobiliary type was found to be more common than the intestinal type. Of a total of 17 cases of the intestinal type, 2 cases were CK7- and CK20+, 4 cases were CK7 + and CK20-, 7 cases were CK7+ and CK20+, and 4 cases were CK7- and CK20-. There was also a total of 20 cases of the pancreaticobiliary type: 1 case for CK7-/CK20+, 14 for CK7+/CK20-, 0 for CK7+/CK20+, and 5 for CK7-/CK20-. In this study, the difference in survival rates according to histologic type was not significant. However, immunohistochemical results, in the case of CK7+/CK20-, were identified as a factor affecting survival in multivariate analysis. The prognosis according to histologic subtype of ampullary adenocarcinoma has also been well described in several previous studies. However, there are few studies that include a comparative analysis according to immunohistochemical markers. Immunohistochemical results are essential for the determination of the histological subtype of ampullary adenocarcinoma. But, expression of immunohistochemical marker and histological type are not always matched. Therefore, examination of prognosis as dependent on immunohistochemical markers may be meaningful for predicting the outcome of ampullary adenocarcinoma. Also, a precise immunochemical study of subtyping may provide a valuable tool for better defining ampullary adenocarcinoma, contributing to more accurate predictions of prognosis [10]. In the present study, the identification of CK7+/CK20- as a factor affecting survival in multivariate analysis confirms its role as a pathological indicator predicting a clinical outcome. This immunohistochemical result deserves further research as a potential marker for selection of patients for adjuvant chemotherapy.

The limitations of this study include its retrospective nature and the limited number of patients due to the rarity of ampullary carcinoma. Also, we did not analyze other immunohistochemical subtypes by testing expression of MUC1, MUC2, etc. Additionally, we could not control for treatment received after resection, such as chemotherapy or concurrent chemoradiotherapy, which may have influenced overall survival.

## Conclusions

In conclusion, our study indicates that advanced T stage is a significant prognostic factor affecting survival, and poor differentiation is a significant prognostic factor affecting disease-free survival in ampullary adenocarcinoma. Also, CK7+/CK20- expression is an independent factor related to overall survival.

## Abbreviations

AoV: Ampulla of Vater
CK7: Cytokeratin 7
CK20: Cytokeratin 20
CA 19–9: Carbohydrate antigen 19–9
CEA: Carcinoembryonic antigen
